# A novel noninvasive method for remote heart failure monitoring: the EuleriAn video Magnification apPLications In heart Failure studY (AMPLIFY)

**DOI:** 10.1038/s41746-019-0159-0

**Published:** 2019-08-21

**Authors:** Freddy Abnousi, Guson Kang, John Giacomini, Alan Yeung, Shirin Zarafshar, Nicholas Vesom, Euan Ashley, Robert Harrington, Celina Yong

**Affiliations:** 1 Division of Cardiovascular Medicine, Department of Medicine, Stanford University Medical Center, Palo Alto, CA USA; 2 Yale School of Medicine, Palo Alto, CA USA; 3 Yale School of Medicine, New Haven, CT USA; 4 Veterans Affairs Palo Alto Medical Center, Palo Alto, CA USA

**Keywords:** Diagnosis, Heart failure

## Abstract

Current remote monitoring devices for heart failure have been shown to reduce hospitalizations but are invasive and costly; accurate non-invasive options remain limited. The EuleriAn Video Magnification ApPLications In Heart Failure StudY (AMPLIFY) pilot aimed to evaluate the accuracy of a novel noninvasive method that uses Eulerian video magnification. Video recordings were performed on the neck veins of 50 patients who were scheduled for right heart catheterization at the Palo Alto VA Medical Center. The recorded jugular venous pulsations were then enhanced by applying Eulerian phase-based motion magnification. Assessment of jugular venous pressure was compared across three categories: (1) physicians who performed bedside exams, (2) physicians who reviewed both the amplified and unamplified videos, and (3) direct invasive measurement of right atrial pressure from right heart catheterization. Motion magnification reduced inaccuracy of the clinician assessment of central venous pressure compared to the gold standard of right heart catheterization (mean discrepancy of −0.80 cm H_2_O; 95% CI −2.189 to 0.612, *p* = 0.27) when compared to both unamplified video (−1.84 cm H_2_O; 95% CI −3.22 to −0.46, *p* = 0.0096) and the bedside exam (−2.90 cm H_2_O; 95% CI −4.33 to 1.40, *p* = 0.0002). Major categorical disagreements with right heart catheterization were significantly reduced with motion magnification (12%) when compared to unamplified video (25%) or the bedside exam (27%). This novel method of assessing jugular venous pressure improves the accuracy of the clinical exam and may enable accurate remote monitoring of heart failure patients with minimal patient risk.

## Introduction

Heart failure is a tremendously morbid, deadly, and costly disease that affects 6.5 million Americans today.^1^ It is now implicated in one in nine deaths in the United States and at least 20% of all hospitalizations among persons older than 65.^1^ It costs the United States $30.7 billion annually, a figure forecasted to increase to $70 billion by 2030.^2^

Much of this economic burden is generated by hospitalizations for heart failure, which represent up to 80% of direct costs, making heart failure readmissions a key target for cost reduction.^2^ Preventing readmissions requires early provider intervention in the ambulatory setting, but the ideal timing and methods to achieve such interventions remain elusive. A critical step to reducing heart failure hospitalizations is identifying which patients will imminently decompensate by evaluating their volume status. While physicians have traditionally relied on the bedside exam of the jugular venous pressure (JVP) to assess volume status, this can be impractical as a primary method for monitoring outpatients. With the recent explosion of telemedicine, the majority of healthcare institutions in the United States employ some form of virtual interaction, paving the way for monitoring tools that can tap this potential.

Invasive ambulatory hemodynamic monitors have shown promise as early-warning systems in heart failure. A variety of devices have been developed and studied, including right ventricular (COMPASS-HF and REDUCE HF), left atrial (HOMEOSTASIS and LAPTOP-HF), and pulmonary artery (CHAMPION) pressure measurement systems.^3–7^ While devices such as the CardioMEMS monitor have been demonstrated to reduce heart failure readmissions, they may not be scalable: they are invasively implanted, costly upfront (approximately $17,000 for a CardioMEMS device), and demand additional personnel and resources to handle the datastream.^8^ Furthermore, their cost-effectiveness remains debatable.^8–12^

In this study, we propose a noninvasive and easily scalable alternative to current invasive remote pressure monitoring systems by combining the bedside examination with modern image processing techniques. Eulerian video magnification is an image processing method by which visually imperceptible periodic motions can be deconstructed and amplified into movements discernible to the naked eye.^13^ We describe the application of Eulerian video magnification to the jugular venous pulse examination and demonstrate its potential as a novel method of noninvasive monitoring of right-sided filling pressures.

## Results

### Participant characteristics

Participant characteristics are presented in Table 1. Among the 59 participants enrolled, 48 (81%) completed the study. The most common reason for study noncompletion was due to cancellation of the right heart catheterization (RHC) procedure (6 patients, 10.2%). The average age of patients who completed the study was 70 years (SD: 8.04 years); 85% of participants were over 65 years old. The average BMI was 30 (SD: 4.87) with 83% of participants qualifying as overweight or obese. Most participants had never smoked (77%), while 15% were former smokers and 8% were current smokers. Mean chest circumference among this sample was 120 cm (SD: 12 cm).

**Table 1:** Patient demographics

	Study completed (*N* = 48)
Age, Mean ± SD (IQR)	69.9 ± 8.04 (67–73)
Age brackets, *n* (%)	
40–64 years	7 (14.6)
65–74 years	31 (64.6)
75+ years	10 (20.8)
BMI, Mean ± SD (IQR)	29.6 ± 4.87 (26.3–32.3)
BMI brackets, *n* (%)	
Underweight	1 (2.1)
Normal weight	7 (14.6)
Overweight	17 (35.4)
Obese	23 (47.9)
Smoker, *n* (%)	
No	37 (77.1)
Former	7 (14.6)
Yes	4 (8.3)
Chest circumference (cm), Mean ± SD (IQR)	119.6 ± 12.01 (112.5–129)

*BMI* body mass index

### Invasive vs. non-invasive measurements

A summary of invasive and noninvasive data are reported in Table 2. On average, participant cardiac output and index were normal at 4.94 L/min and 2.36 L/min/m^2^, respectively. The average right atrial pressure (RAP) by right heart catheterization was 8.56 cm H_2_O (IQR 5–10). Similarly, average JVP was 7.11 cm H_2_O (IQR 5–8) when measured at bedside, 7.8 cm H_2_O (IQR 6.2–8.4) when measured from unamplified video, and 8.8 cm H_2_O (IQR 7.2–10) when measured from motion-amplified video.

**Table 2:** JVP and RHC measurements. Cardiologist mean pressure measurements are reported in cm H_2_O except as otherwise noted

	Study completed (*N* = 48)	
	*N*	Mean ± SD (IQR)
RHC		
Cardiac output	48	4.94 ± 0.92 L/min (4.25–5.50)
Cardiac index	48	2.36 ± 0.34 L/min/m^2^ (2.18–2.56)
Heart rate	48	65.73 ± 12.53 beats/min (59–73.25)
PAP (mean)	48	21.02 ± 7.47 mmHg (16–24)
PCWP	48	15.17 ± 7.97 mmHg (9.75–19.5)
RAP	48	8.56 ± 5.09 cm H_2_O (5–10)
Bedside physician exams		
JVP: 1st physician exam	48	7.06 ± 2.85 (5–8)
JVP: 2nd physician exam	48	6.52 ± 3.14 (5–7)
JVP (mean)	48	7.11 ± 3.21 (5–8)
Videos: Unamplified		
JVP: Cardiologist 1	48	7 ± 2 (5–10)
JVP: Cardiologist 2	48	9 ± 2 (7–11)
JVP: Cardiologist 3	48	9 ± 3 (7–11)
JVP: Cardiologist 4	48	8.3 ± 3.4 (6–10)
JVP: Cardiologist 5	48	7 ± 1 (6–8)
JVP: Cardiologist 6	48	9 ± 3 (6–10)
JVP: Cardiologist 7	48	5 ± 4 (2–5)
JVP: Cardiologist 8	48	6 ± 3 (5–7)
JVP: Cardiologist 9	48	10 ± 2 (8–10)
JVP (mean)	48	7.8 ± 2 (6.2–8.4)
Videos: Amplified		
JVP: Cardiologist 1	48	7 ± 3 (5–10)
JVP: Cardiologist 2	48	9 ± 2 (7–11)
JVP: Cardiologist 3	48	11 ± 3 (8–13)
JVP: Cardiologist 4	48	9.4 ± 3.8 (7–11)
JVP: Cardiologist 5	48	8 ± 2 (7–9)
JVP: Cardiologist 6	48	10 ± 3 (8–12)
JVP: Cardiologist 7	48	8 ± 4 (5–10)
JVP: Cardiologist 8	48	7 ± 3 (5–9)
JVP: Cardiologist 9	48	11 ± 3 (9–12)
JVP (mean)	48	8.8 ± 2.2 (7.2–10)

*JVP* jugular venous pressure, *RHC* right heart catheterization, *RAP* right atrial pressure, *PAP* pulmonary artery pressure, *PCWP* pulmonary capillary wedge pressure, *IQR* interquartile range

All noninvasive modalities yielded JVPs lower than the corresponding invasive right atrial pressures, though the magnitude varied depending on the modality (Table 3). When measured at the bedside, JVP was on average 2.90 cm H_2_O lower than corresponding right heart catheterization measurements (95% CI −4.33 to 1.40, *p* = 0.0002). This discrepancy narrowed when measured via unamplified video (−1.84 cm H_2_O; 95% CI −3.22 to −0.46, *p* = 0.0096). After motion magnification, this discrepancy was no longer significant (−0.80 cm H_2_O; 95% CI −2.189 to 0.612, *p* = 0.27).

**Table 3:** Comparison of means between clinically and invasively measured jugular venous pressure

Measurement differences	Mean difference (cm H_2_O)	95% CI for mean difference	Wilcoxon signed rank *p* value
Bedside exam vs. RHC	−2.90	−4.33 to −1.40	0.0002
Unamplified video vs. RHC	−1.84	−3.22 to −0.46	0.0096
Amplified video vs. RHC	−0.80	−2.18 to 0.61	0.27

*CI* confidence interval, *RHC* right heart catheterization

**Fig. 1:**
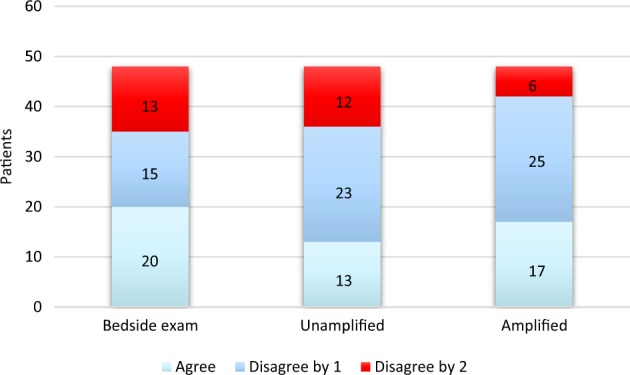
Agreement with right heart catheterization by assessment type. “Agreement” with right heart catheterization was achieved if clinicians characterized the JVP similarly (e.g., both were “elevated”); “disagree by 1” was noted where a one-category discrepancy existed (e.g., one was “normal” and the other was “borderline”); and “disagree by 2” was noted if there was a two-category discrepancy (e.g., one was “normal” and the other was “elevated”)

To better understand the practical clinical utility of the video magnification, we compared the noninvasive to the invasive measurements by characterizing them qualitatively. Each invasive and noninvasive measurement was designated “normal” if it was less than or equal to 7 cm H_2_O, “borderline” if greater than 7 but less than or equal to 10 cm H_2_O, or “elevated” if greater than 10 cm H_2_O. “Agreement” with right heart catheterization was achieved if both categories were the same (e.g., both were “elevated”); partial disagreement was noted if there was a one-category discrepancy (e.g., one was “normal” and the other was “borderline”); and complete disagreement was noted if there was a two-category discrepancy (e.g., one was “normal” and the other was “elevated”). These pooled data are presented in Fig. 1. Complete disagreement was found in 27% of bedside assessments, 25% of unamplified video assessments, and only 12% of amplified video assessments. Neither bedside exam nor unamplified video assessments skewed in a statistically significant fashion towards “agreement” or “disagreement” with right heart catheterization (*p* = 0.44 and *p* = 0.10, respectively; Table 4). In contrast, assessments made with amplified video more often agreed or partially disagreed (*p* = 0.0034) than the other noninvasive modalities.

**Table 4:** Accuracy of JVP characterization compared to right heart catheterization

Category^a^	Observed	Expected	Pearson’s *χ*^2^	*p* value
Bedside exam				
Agree	20	16	1.625	0.44
Disagree by 1	15	16		
Disagree by 2	13	16		
Unamplified				
Agree	13	16	4.625	0.10
Disagree by 1	23	16		
Disagree by 2	12	16		
Amplified				
Agree	17	16	11.375	**0.0034**
Disagree by 1	25	16		
Disagree by 2	6	16		

*JVP* jugular venous pressure

^a^“Agreement” achieved if the assessment categorization was the same as right heart catheterization (e.g., both “borderline”); “disagree by 1” noted if there was a one-category discrepancy (e.g., “normal” and “borderline”); “disagree by 2” noted if there was a two-category discrepancy (e.g., “normal” and “elevated”). Expected values assume a random frequency distribution across categories

Bold value indicate statistical significance

## Discussion

The AMPLIFY pilot study suggests that computerized motion amplification can improve the accuracy of clinical bedside JVP measurement. Historically, studies of the bedside exam have reported variable accuracy, and some suggest that it tends to underestimate the central venous or right atrial pressure.^14,15^ Our data confirm this tendency. Furthermore, our data suggest that the bedside exam is inaccurate at characterizing right-sided filling pressures: 27% of characterizations made at the bedside by two independent cardiologists were completely erroneous, which may reflect the high proportion of obesity in this study. Despite the challenging population, motion magnification resulted in less central venous pressure discrepancy with right heart catheterization as compared to bedside or unamplified video assessments. Perhaps more relevantly, it reduced significant categorical disagreements with right heart catheterization compared to the other noninvasive modalities.

Since its first description in 2012, Eulerian phase-based motion amplification has been re-tooled for a variety of purposes, such as measuring heart rate by amplifying the imperceptible color shifts of the skin with each pulse^13^ or localizing and labeling major blood vessels during surgeries in real time.^16^ This method is the first published application of Eulerian phase-based motion amplification to heart failure diagnostics.

Perhaps most importantly, the AMPLIFY ambulatory heart failure monitoring strategy has the potential to expand modern telehealth capabilities. Telehealth adoption has been estimated to exceed 60% across all U.S. healthcare institutions, and virtual interactions (e.g. video, telephone, e-mail) already outnumber in-person visits in some healthcare systems.^17^ With that in mind, we designed AMPLIFY to emulate the telehealth setting: all video assessments were completed by cardiologists who never made physical contact with study participants. Unlike current invasive monitoring tools, which generate overwhelming amounts of data that do not integrate into existing electronic health record systems, the on-demand nature of motion-amplified monitoring should yield a more manageable datastream, generated at clinically important time points. Finally, as a software-based technique, motion amplification has great potential for scalability as it requires no specialized equipment beyond what is already available in many telehealth ecosystems. Given our use of commercially available video equipment, we speculate that this technology could also be eventually adapted for use with video equipment available on most modern mobile phones.

AMPLIFY has several limitations. As a pilot study, the cohort is small and derived from a predominantly male Veterans Affairs population, and the results should not be generalized beyond the scope of the study. AMPLIFY was intentionally conducted on an outpatient population to emulate the telehealth setting and therefore does not apply to inpatient monitoring. The study was not designed to examine clinical outcomes such as heart failure decompensations or cost-effectiveness. From a technical standpoint, the method itself is reliant on detecting changes in local contrast and may have theoretical limitations when applied to patients with darker skin tones, though our study did capture a variety of skin tones. Finally, the clinical assessment of JVP in general can be inaccurate when it is very high (e.g., above the earlobe); as such, we do not consider it a quantitative technique in its current form.

The next steps will be to refine, automate, and implement this novel technique in the telemedicine setting to compare it with modern invasive monitoring systems on a larger scale. Visual amplification techniques are heavily reliant on image contrast, and improvements to acquisition technique, such as using off-camera point light sources to improve local contrast and signal-to-noise yield, may improve accuracy. This may be especially relevant to patients with darker skin tones and naturally lower tonal contrast. Furthermore, standardizing acquisition position, scale, and distance from the patient, as well as identification of landmarks from which JVP is clinically measured (e.g., clavicle or the sternal angle) are potential targets for optimization. In the future, advances in mobile hardware capabilities may improve clinical usability by allowing for real-time visualization of the jugular venous pulse (so-called “augmented reality”). Finally, automation of the measurement process using computer vision techniques could further decrease the need for human supervision and improve cost-efficiency.

In conclusion, AMPLIFY serves as a pilot to demonstrate the potential utility and scalability of motion magnification for providing clinically relevant remote monitoring of patients with heart failure.

## Methods

The AMPLIFY study was a pilot clinical trial evaluating the efficacy of using video image processing as a novel method for measuring JVP. This study was approved by both the Stanford Institutional Review Board and the Veterans Affairs Research and Development Board. Written informed consent was obtained from each participant.

### Participants

Eligible participants were screened through the VA electronic medical record system. Patients were eligible for inclusion if they were over 18 years of age and were scheduled to undergo right heart catheterization for any indication at the Palo Alto Veterans Affairs Hospital. Exclusion criteria included existing right internal jugular vascular access, ventilatory support or airway/cervical deformity interfering with neck visualization, inability to tolerate lying at a 30 or 45-degree angle, or inability to lie still for the duration of the video recording.

### Procedure

**Fig. 2:**
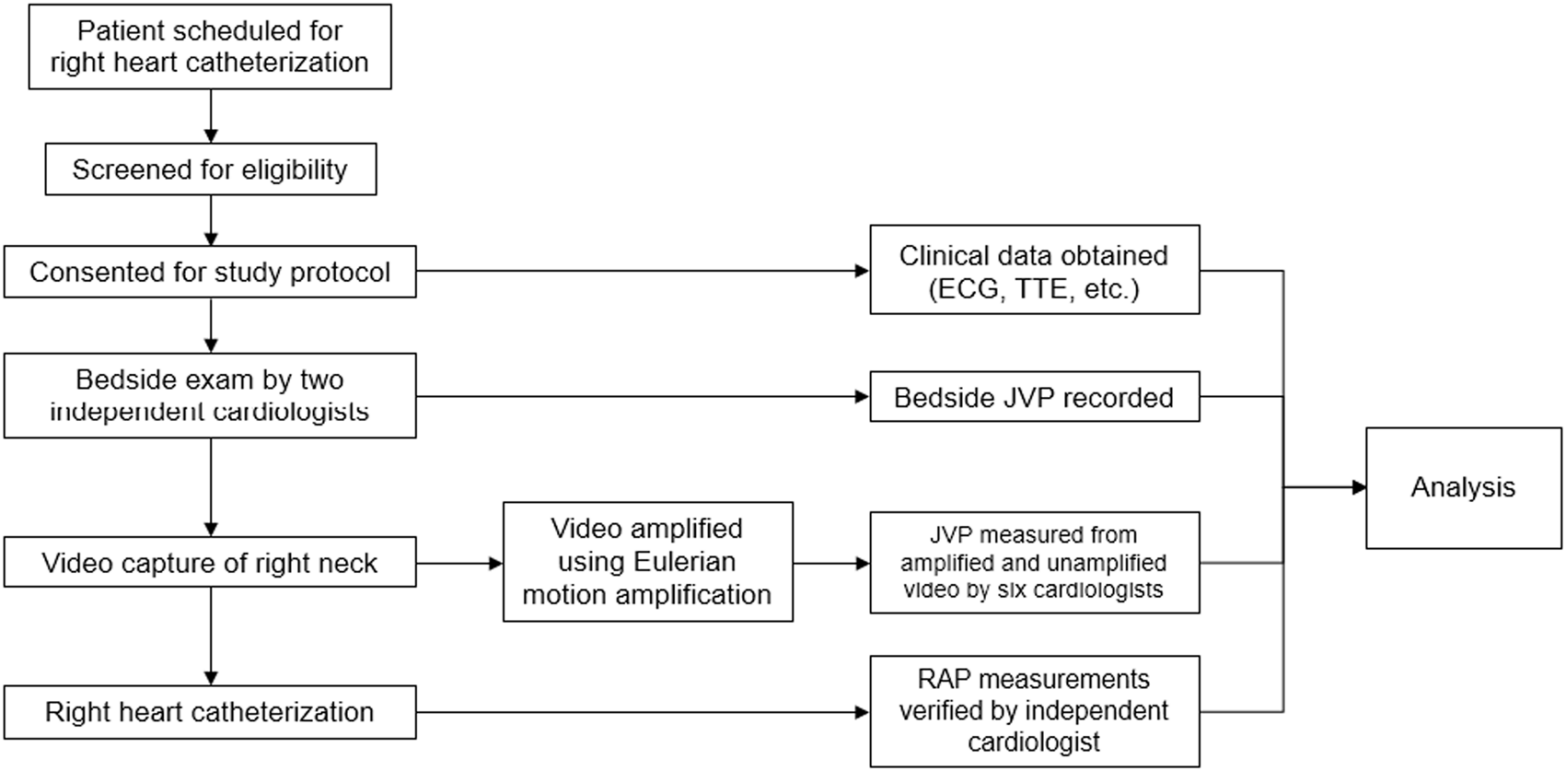
AMPLIFY protocol. ECG electrocardiogram, TTE transthoracic echocardiogram, JVP jugular venous pressure, RAP right atrial pressure

The AMPLIFY protocol is outlined in Fig. 2. After potential participants were screened for eligibility, informed consent was obtained and participants were assigned an anonymous study identification number.

Using commercially available video and lighting equipment, two 60-s video clips of the right side of the participant’s neck were acquired: one with the participant lying on a standard wedge pillow at a 45° angle, and the second while sitting at a 90° angle. During all video acquisition, the patient was monitored with a continuous three-lead electrocardiogram (right upper chest, left upper chest, one leg) with a commercially available portable ECG machine (Heal Force PC-80B). The acquisition system consisted of a commercial digital camera connected to a laptop PC. The camera system consisted of a Basler aCA1920-155uc (Sony IMX174 global shutter CMOS sensor) with a Kowa LM25HC 1″ 25 mm F1.4 lens. A battery-powered LED light source was mounted above the camera facing the same direction to provide illumination. A laptop computer running custom software streamed uncompressed, 1920 × 1280, 8-bit RGB at 155 frames per second to a solid-state drive. The acquisition system was stabilized on a tripod cart to reduce artifact from translational and rotational vibrations.

Two cardiologists independently performed bedside examinations of each participant’s JVP and recorded their measurements prior to right heart catheterization. Clinicians qualitatively assessed venous pressure by estimating the orthogonal height of the JVP column, which appears as a subtle, transient movement of the skin. Copies of the pre-catheterization ECG and the participant’s most recent echocardiogram report within the last year were collected. Upon completion of right heart catheterization, copies of all pressure tracings were obtained, from which pressure measurements were extracted by an independent cardiologist.

**Fig. 3:**
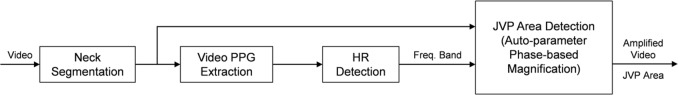
Video stream magnification procedure. PPG photoplethysmogram

The de-identified video recordings were then sent to Google LLC for image processing. The video streams were processed as shown in Fig. 3. First, the neck region was segmented using a weighted Gaussian mixture model. A video photoplethysmogram (vPPG) was then extracted to determine the heart rate, which was used in the later motion magnification block to set the frequency bands of interest.

**Fig. 4:**
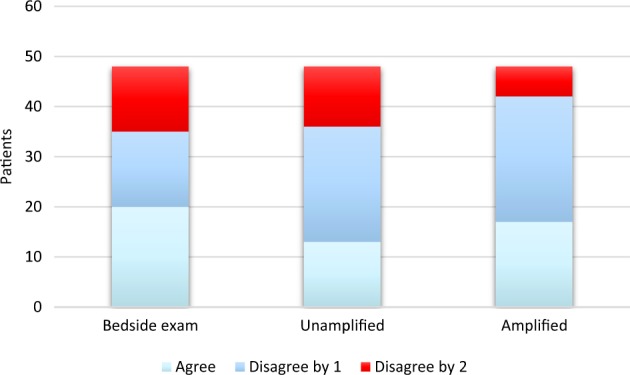
Video magnification screen capture. Still screen capture from unamplified video (left) and amplified video (right). Though easier to appreciate while in motion (Supplemental Videos 1 and 2), exaggerated pulsatile distension of the internal jugular vein is visible as a motion blur-like artifact up to the black arrow. Written informed consent allowing publication of the participant’s images was obtained

Eulerian phase-based video magnification was then used to amplify the pulsations of the right internal jugular vein as described in Wu et al.^13^ and Wadhwa et al.^18^ The basic principle of the approach is to decompose a standard video sequence into a time series of color values at a given pixel. This time series is then filtered for a prespecified band of temporal frequencies (in this application, a range including normal resting human heart rates); the resultant signal is then amplified by a specified magnification factor and reconstituted back into video format. Our code was built off the publicly available MATLAB code; signal processing was performed by commercially available personal computers. A sample video is included in the supplementary materials (Supplementary Video 1 demonstrates unamplified and amplified video); a representative screen capture from the video is shown in Fig. 4.

After image processing, both the unamplified and amplified videos were assessed by nine cardiologists. The cardiologists were blinded with respect to all patient data, including the right heart catheterization results, and were not involved with any other part of the study. The cardiologists were instructed to approximate the JVP based on both the unamplified and amplified video streams without the use of measuring aids to reflect real-world practice; scale was inferred visually using anatomic landmarks.

#### Measures

Demographic (age, smoking history) and anthropometric (height, weight, chest circumference) data were collected from each participant during the study visit. Participants’ body mass indices were calculated from the height and weight and categorized using the CDC definitions for underweight (<18.5), normal weight (18.6–24.9), overweight (25–29.9), and obese (≥30).

Each bedside cardiologist’s assessment included an estimation of the JVP at bedside (reported in centimeters of water) and the angle at which it was assessed (Supplementary Table 1). Data obtained from the right heart catheterization tracings included pressure values for the right atrium, right ventricle, pulmonary capillary wedge pressure, pulmonary artery, as well as cardiac output, cardiac index, intraprocedural heart rate, and a record of the sedative medications given. Cardiologist assessment of the unamplified and amplified jugular vein videos reported estimated JVP in centimeters of water (Supplementary Table 2).

To be included in the final analysis, participants were required to have completed (1) right heart catheterization, (2) bedside assessment, and (3) video assessment.

### Statistical analysis

JVP assessments from the noninvasive techniques and RHC measurements were descriptively summarized for all participants who completed the study. The average of the bedside JVP assessments was calculated for each participant to obtain an averaged JVP to compare against the RHC RAP. The average JVP assessed by video (unamplified and amplified) was also compared against the RHC RAP. To translate the measurements to clinically actionable categories, we converted them to “normal,” “borderline,” or “elevated” if they were less than or equal to 7 cm H_2_O, greater than 7 but less than or equal to 10 cm H_2_O, or greater than 10 cm H_2_O, respectively.^19–22^ Mean differences and 95% CI of the mean difference was calculated between each of the averaged JVPs and RHC right atrial pressure. All analyses were conducted using SAS 9.3 (Cary, NC).

### Reporting summary

Further information on research design is available in the Nature Research Reporting Summary linked to this article.

## Supplementary information

Reporting Summary Checklist

Supplementary Information.

Supplemental Video 1

## Data Availability

The hemodynamic data that support the findings of this study are anonymized and presented in the Supplementary Information. Additional video data that support the findings of this study are available on request from the corresponding author (C.Y.). The video data are not publicly available to minimize any exposure of potentially sensitive material.

## Supplementary information

**Supplementary information** accompanies the paper on the *npj Digital Medicine* website (10.1038/s41746-019-0159-0).

## Code availability

The code employed to process the video streams is publicly available at http://people.csail.mit.edu/mrub/vidmag/#code.
