# Supplementary material for: A novel noninvasive method for remote heart failure monitoring: the EuleriAn video Magnification apPLications In heart Failure studY (AMPLIFY)
Source: NPJ Digit Med. 2019 Aug 21;2:80. doi: 10.1038/s41746-019-0159-0 (PMC6704101; doi:10.1038/s41746-019-0159-0)
Supplement: Supplementary file 2 — Supplementary Information. [file 41746_2019_159_MOESM2_ESM.pdf]

Supplementary Table 1. *Cardiologist bedside exam findings.* JVP = jugular venous pressure. NC = patient enrolled but right heart catheterization aborted or bedside assessment not completed.

Patient ID	Invasive right atrial pressure (mmHg)	Bedside JVP Cardiologist 1(cm H_2O)	Bedside JVP Cardiologist 2 (cm H_2O)
**1**	5	10	5
**2**	9	15	15
**3**	7	5	6
**4**	3	6	10
**5**	8	6	6
**6**	9	6	7
**7**	4	5	5
**8**	7	5	6
**9**	11	8	9
**10**	8	5	6
**11**	3	7	0
**12**	7	5	5
**13**	10	6	6
**14**	10	7	6
**15**	5	5	6
**16**	8	5	6
**17**	13	10	4
**18**	15	12	15
**19**	7	8	7
**20**	2	6	NC
**21**	3	10	7
**22**	22	15	6
**23**	8	8	8
**24**	8	5	7
**25**	3	6	5
**26**	6	5	7
**27**	8	6	7
**28**	10	5	5
**29**	5	6	5
**30**	8	10	2
**31**	4	8	3
**32**	17	15	15
**33**	4	5	6
**34**	10	4	10
**35**	9	5	0
**36**	5	7	5
**37**	11	8	8
**38**	16	8	NC
**39**	11	7	6
**40**	11	7	6
**41**	8	6	NC
**42**	10	1	NC
**43**	6	5	6
**44**	5	6	6
**45**	6	8	6
**46**	30	5	4
**47**	12	10	11
**48**	4	6	6

Supplementary Table 2. *Cardiologist video exam findings.* All data presented in cm H_2O. NC = assessment not completed.

ID	Cardiologist 1		Cardiologist 2		Cardiologist 3		Cardiologist 4		Cardiologist 5		Cardiologist 6		Cardiologist 7		Cardiologist 8		Cardiologist 9	
	Raw	Amplified	Raw	Amplified	Raw	Amplified	Raw	Amplified	Raw	Amplified	Raw	Amplified	Raw	Amplified	Raw	Amplified	Raw	Amplified
**1**	8	8	9	9	6	8	7	7	7	7	6	8	7	9	5	5	9	10
**2**	11	12	12	10	13	13	11	11	8	8	13	11	14	15	10	10	12	18
**3**	5	5	7	7	7	8	6	6	6	6	NC	NC	5	6	4	4	8	8
**4**	7	7	10	10	8	11	8	9	7	7	9	9	4	7	7	7	11	11
**5**	7	7	10	12	7	7	7	7	NC	NC	7	8	2	5	5	5	9	9
**6**	5	5	7	7	12	12	6	6	6	7	6	6	5	5	5	5	8	9
**7**	5	5	7	7	5	7	6	6	6	6	5	6	2	3	4	4	8	8
**8**	7	7	NC	7	9	9	7	8	9	10	7	7	2	5	8	6	11	11
**9**	10	10	11	13	11	13	10	12	10	10	10	11	5	6	5	5	13	14
**10**	5	5	12	12	8	11	6	7	6	7	5	6	5	8	5	5	9	11
**11**	5	5	8	10	11	7	5	5	7	8	NC	NC	2	5	4	4	8	9
**12**	6	6	8	8	8	9	6	7	6	9	7	8	5	6	4	5	8	9
**13**	8	8	10	10	10	13	9	10	8	8	7	10	7	10	5	5	9	11
**14**	7	7	7	9	15	15	8	7	8	10	6	8	6	10	5	9	10	10
**15**	5	5	7	7	10	10	6	8	5	7	9	10	5	5	4	4	8	9
**16**	5	5	11	14	10	15	6	7	9	12	13	15	3	6	8	8	9	18
**17**	10	10	7	7	5	8	7	7	7	9	5	7	0	4	4	4	10	14
**18**	5	5	15	15	8	9	6	7	8	8	6	8	2	5	4	4	9	10
**19**	6	6	8	7	7	14	7	9	7	10	9	11	3	5	4	4	11	13
**20**	5	5	7	7	7	10	7	8	6	8	8	8	2	5	5	6	9	10
**21**	8	8	11	11	13	13	10	12	8	9	12	10	7	8	8	9	13	14
**22**	6	6	10	14	15	15	17	17	8	10	15	15	3	4	7	9	10	16

**23**	10	10	11	10	12	15	10	8	10	8	10	8	14	15	10	10	14	16
**24**	12	12	7	11	7	12	6	12	6	8	5	18	3	15	5	7	8	11
**25**	10	10	11	11	10	13	11	11	7	8	12	12	5	12	14	14	13	13
**26**	5	5	9	9	7	12	7	15	7	6	13	15	4	10	6	9	9	11
**27**	5	5	7	7	7	9	6	6	6	6	7	8	5	6	6	6	9	10
**28**	6	6	7	9	6	7	7	8	6	6	8	11	3	4	5	5	8	9
**29**	5	5	7	7	5	5	5	5	7	7	8	8	0	2	6	6	8	8
**30**	5	5	9	9	7	7	6	6	6	6	6	6	2	4	6	6	8	9
**31**	11	11	11	13	12	14	10	12	8	9	10	12	15	15	14	14	12	13
**32**	15	15	15	15	15	15	18	20	7	6	18	18	2	20	14	14	8	8
**33**	7	7	8	8	7	7	7	7	6	6	6	6	5	5	5	6	9	9
**34**	5	5	7	7	6	12	5	18	6	6	8	17	5	6	5	7	9	9
**35**	8	8	9	9	9	12	7	8	7	8	8	9	8	10	7	8	11	12
**36**	10	10	12	12	9	10	11	11	9	9	13	13	15	16	12	12	10	12
**37**	10	10	8	10	7	7	9	10	7	8	7	9	5	5	5	5	9	8
**38**	12	12	7	7	13	15	7	9	8	9	10	12	7	15	10	10	8	9
**39**	7	7	7	7	7	7	18	18	8	8	5	6	5	5	5	5	9	9
**40**	6	6	7	7	8	9	7	9	7	8	7	8	0	5	4	4	10	10
**41**	6	6	7	9	5	10	6	7	7	6	12	10	2	5	5	6	8	11
**42**	10	10	8	8	12	12	13	12	10	11	8	13	0	7	9	9	10	12
**43**	7	7	7	7	6	6	6	6	6	7	6	6	3	4	4	4	9	9
**44**	10	10	7	9	10	10	10	9	8	8	8	8	0	7	6	6	11	11
**45**	10	5	14	14	13	13	17	18	12	14	14	14	15	12	12	12	13	13
**46**	5	5	7	7	5	7	NC	NC	6	6	NC	NC	0	3	4	4	8	9
**47**	5	5	7	7	8	11	6	7	6	7	6	7	2	4	5	5	9	10

**48**	8	8	8	9	8	10	7	7	8	7	7	8	5	7	6	6	8	8

Supplementary Video 1. Example of unamplified (left) and amplified (right) video capture. Written informed consent allowing publication of the participant's images was obtained.
